# Supplementary figures and images for: Avian Influenza A Viruses Reassort and Diversify Differently in Mallards and Mammals
Source: Viruses. 2021 Mar 19;13(3):509. doi: 10.3390/v13030509 (PMC8003500; doi:10.3390/v13030509)

# Supp Fig 1

## A

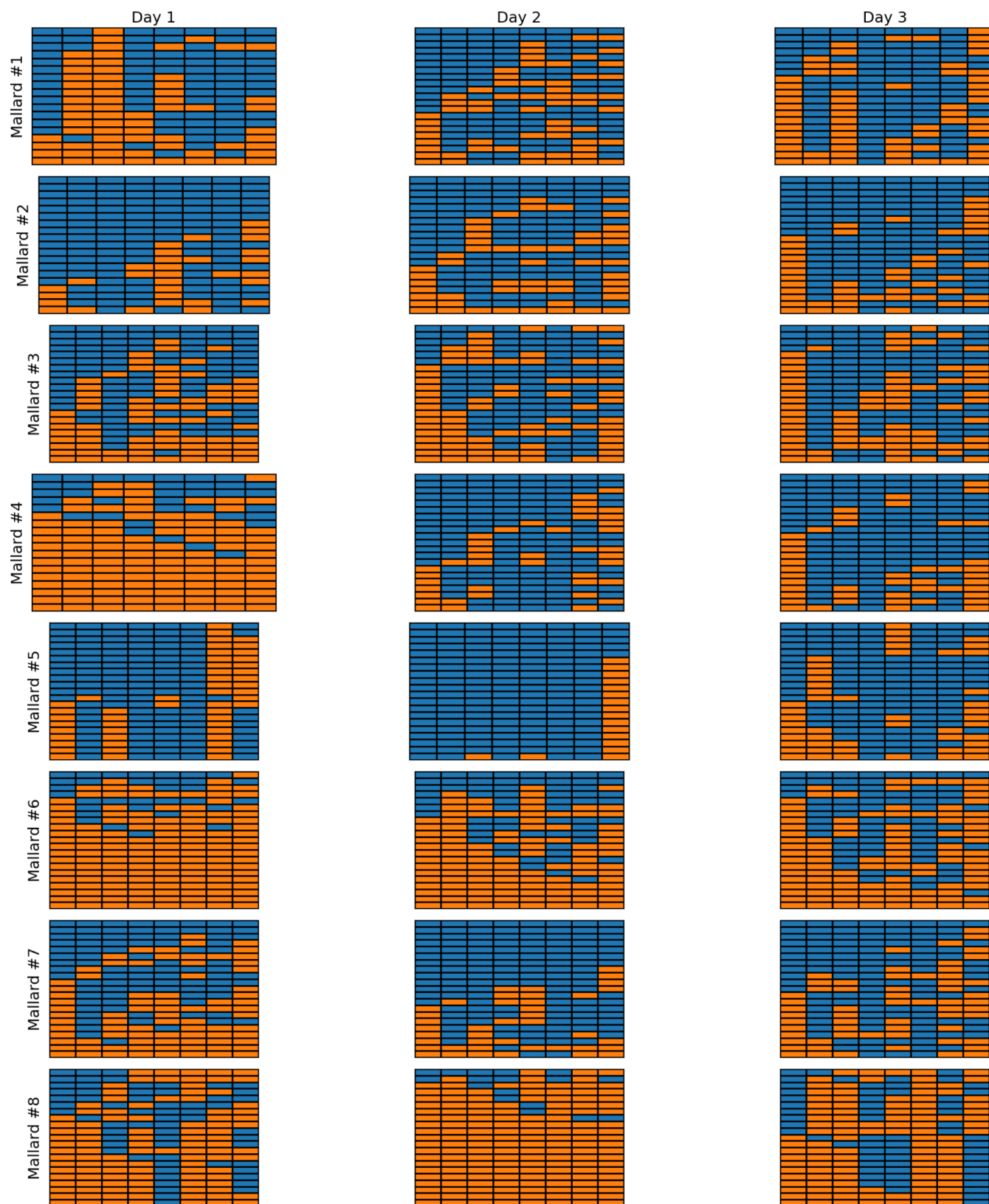

# Supp Fig 1

## B

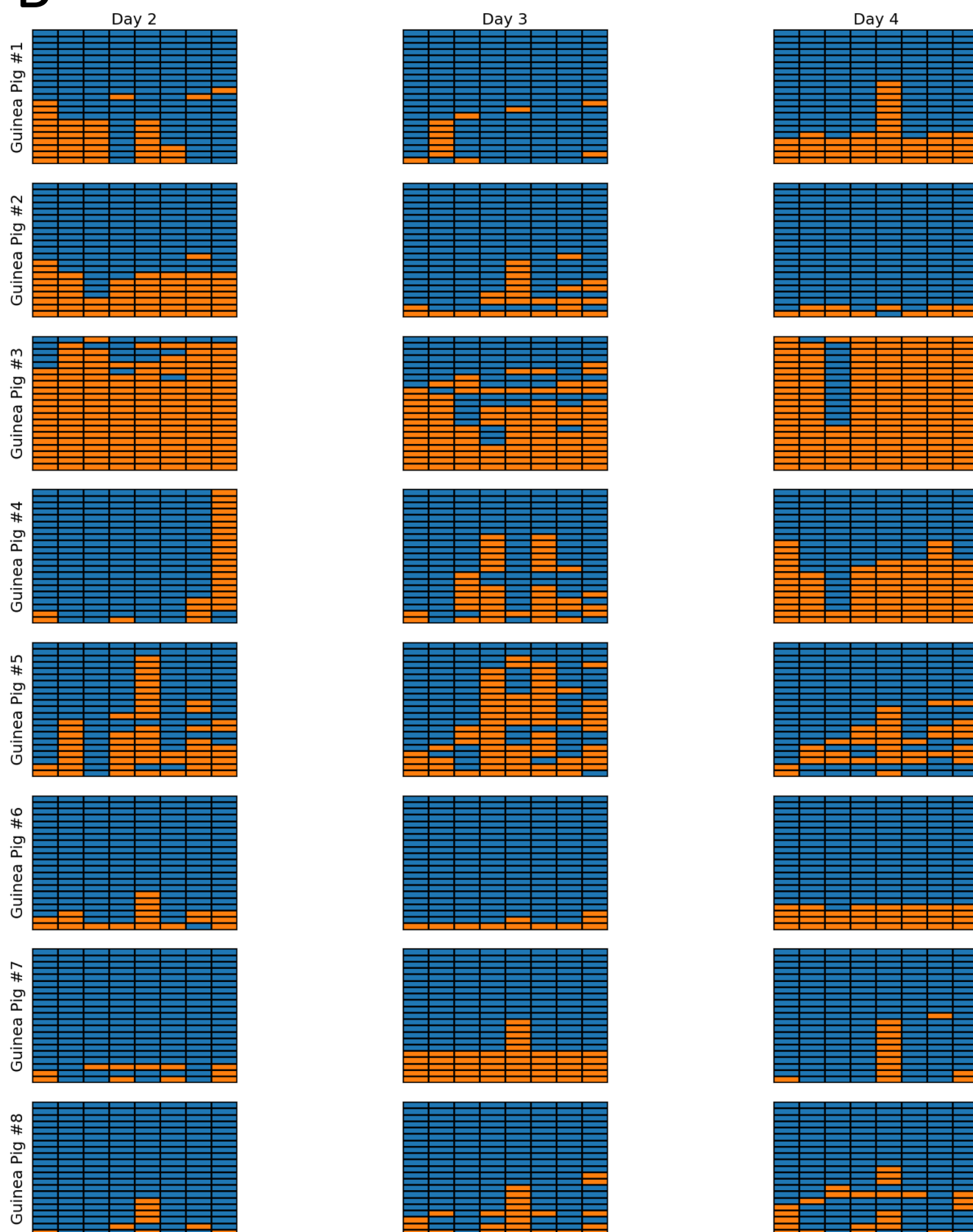

Supp Fig 2

A

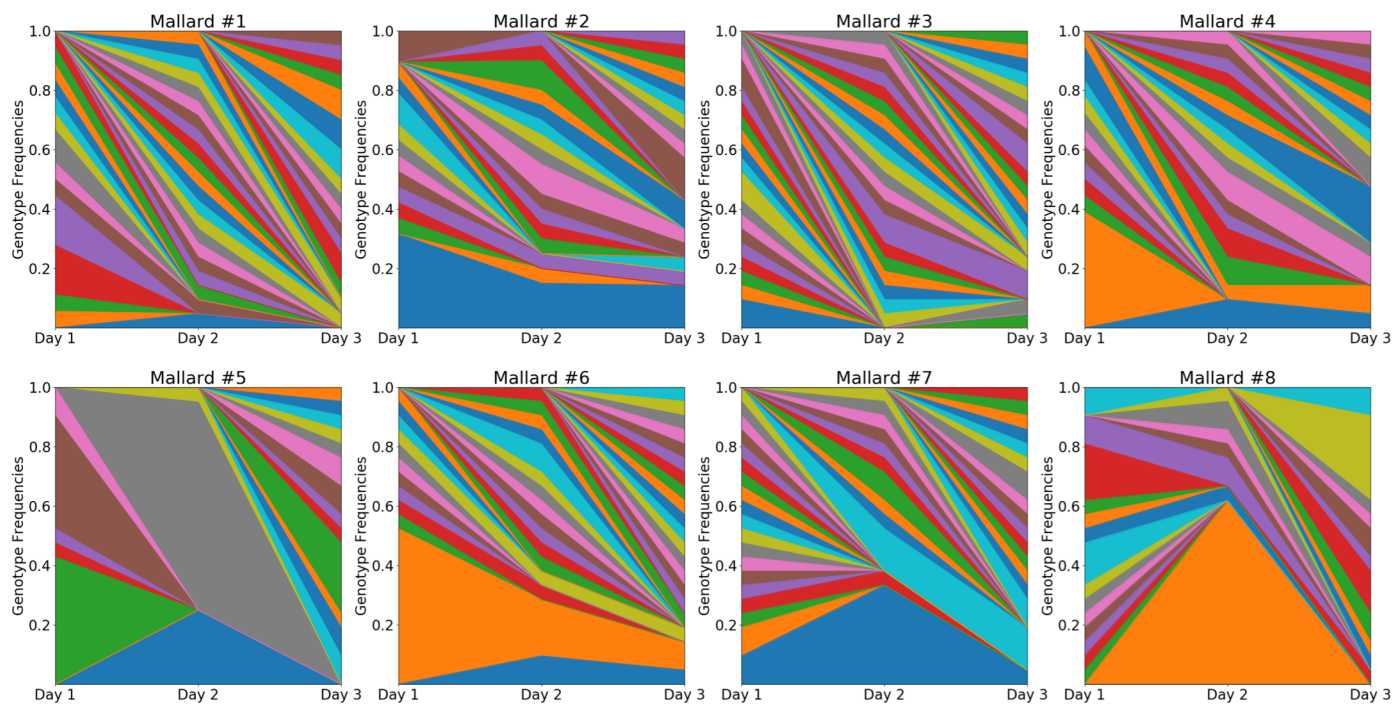

B

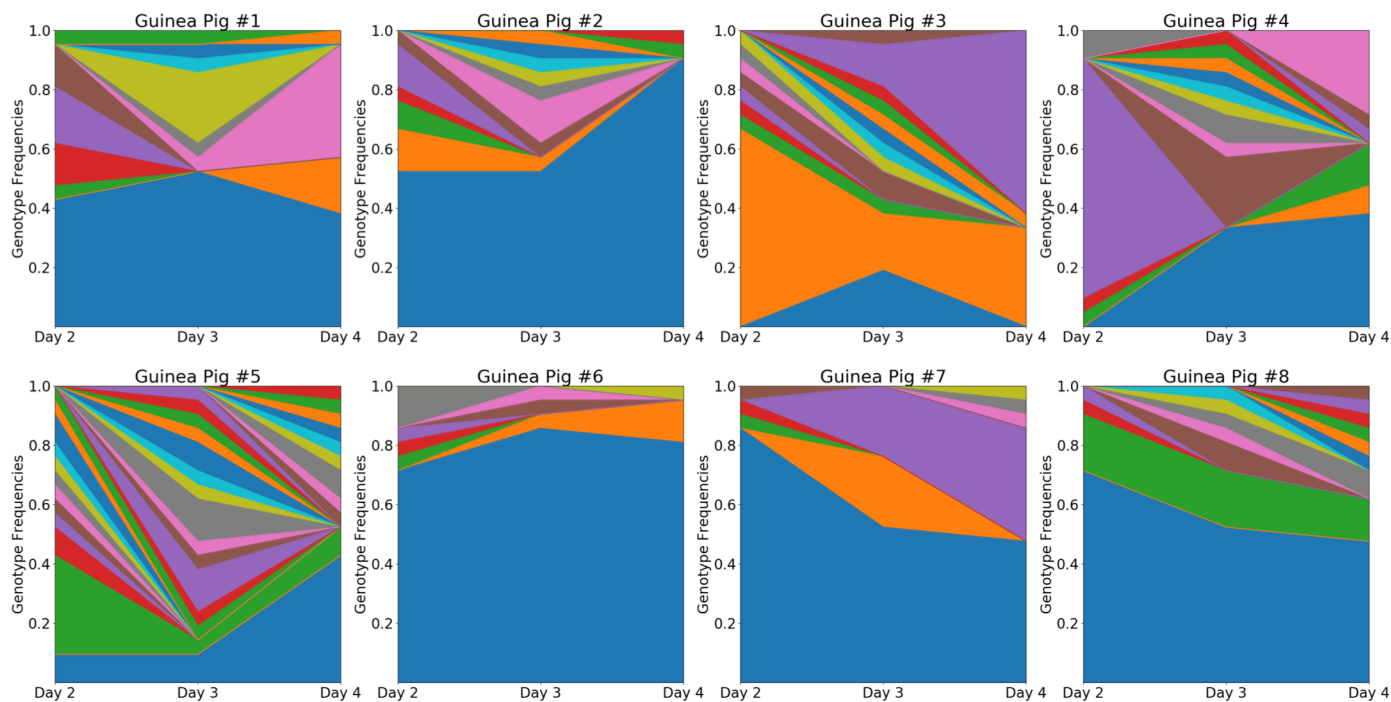

Supplement: Supplementary file 1 [file viruses-13-00509-s001.pdf]
